# Effects of metal oxide inhalation on the transcription of some hormone receptors in the brain, examined in an in vivo mouse model

**DOI:** 10.1007/s11356-024-34425-0

**Published:** 2024-08-12

**Authors:** David Sandor Kiss, Istvan Toth, Tibor Bartha, Akos Jerzsele, Attila Zsarnovszky, Erzsebet Pasztine Gere, Silvia Ondrasovicova, Petra Varro, Csaba Kovago

**Affiliations:** 1 Department of Physiology and Biochemistry, University of Veterinary Medicine, Budapest, Hungary; 2 Department of Pharmacology and Toxicology, University of Veterinary Medicine, Budapest, Hungary; 3 Department of Physiology and Animal Health, Hungarian University of Agricultural and Life Sciences, Godollo, Hungary; 4 Agribiotechnology and Precision Breeding for Food Security National Laboratory, Institute of Physiology and Nutrition, Department of Physiology and Animal Health, Hungarian University of Agricultural and Life Sciences, Godollo, Hungary; 5 Department of Biology and Physiology, University of Veterinary Medicine and Pharmacy in Košice, Košice, Slovakia; 6 Department of Physiology and Neurobiology, Institute of Biology, Eötvös Loránd University, Budapest, Hungary

**Keywords:** Welding, Cerebellum, Hypothalamus, Olfactory bulb, Estrogen receptors, Thyroid receptors, Peroxisome proliferator-activated receptor-gamma, Neuroinflammation

## Abstract

Respirable metal oxide nanoparticles in welding fumes pose significant health risks upon inhalation, potentially leading to neurodegenerative diseases. While the exact mechanisms remain unclear, it is evident that metal oxide nanoparticles can disrupt cellular functions, including metabolism and inflammatory responses after crossing the blood–brain barrier (BBB). Our study investigates the impact of manual metal arc welding fumes on hormone receptor transcription in an in vivo mouse model. After collecting samples from six different brain regions at 24 and 96 h upon exposure, we focused on expression levels of estrogen receptors (ERs), thyroid hormone receptors (TRs), and peroxisome proliferator-activated receptors (PPARs) due to their roles in modulating neuroprotective responses and neuroinflammatory processes. Analysis revealed differential susceptibility of brain regions to hormonal disruption induced by welding fumes, with the hypothalamus (HT) and olfactory bulb (OB) showing prominent changes in receptor expression. Considering ERs, 24 h sampling showed an elevation in OB, with later increases in both ERα and ERβ. HT showed significant ERβ change only by 96 h. TRs mirrored ER patterns, with notable changes in OB and less in HT. PPARγ followed TR trends, with early upregulation in HT and downregulation elsewhere. These findings suggest a compensatory response within the CNS aimed at mitigating neuroinflammatory effects, as evidenced by the upregulation of ERβ, TRα, and PPARγ. The coordinated increase in ERs, TRs, and PPARs in the hypothalamus and olfactory bulb also highlights their potential neuroprotective roles in response to welding fume exposure. Our results also support the theory of metal oxide penetration to the CNS via the lungs-blood-BBB pathway, making HT and OB more vulnerable to welding fume exposure.

## Introduction

Welding is a globally widespread industrial process that involves diverse technologies to create solid joints between metal parts by heating them to their melting points. The method generates a fume containing a complex mixture of metals, metallic oxides, silicates, and fluorides posing inhalation risks in the absence of adequate extraction devices (Antonini 2003; Graczyk et al. 2016). Our research focuses on the effects of fumes produced using the manual metal arc (MMA) welding technique with coated electrodes.

The initial toxic effects of incorporated metals and metal oxides (from welding fume) typically manifest as local symptoms at the place of primary contact (mostly affecting the gastrointestinal tract or airways) (Witkowska et al. 2021). Inhalation of these particles leads to lung problems such as bronchial inflammation, reduced lung capacity, an increased prevalence of inflammatory diseases, and potentially contributing to the development of lung tumors (Riccelli et al. 2020; Samulin Erdem et al. 2020; Kővágó et al. 2022). In the primary contact area, metals and metal oxides can be absorbed so they might reach other parts of the body, potentially culminating even in systemic inflammation (Shen et al. 2018). Although their toxicity is dose-dependent, long-term exposure (even in low doses) always causes clinical signs (Hagberg et al. 1986).

The key factor determining the impact of welding fume is its metal and metal oxide composition. In the MMA technique, manganese (Mn) and iron (Fe) particles, and their oxides, are especially significant, though their concentrations vary based on the equipment and materials used (Zimmer and Biswas 2001). These particles can cross the blood–brain barrier (BBB); thus, prolonged inhalation of even small amounts leads to neurological symptoms (Antonini 2003; Behera et al. 2023). The exact relationship and the neurophysiological pathomechanisms remain unclear as most of the existing literature discusses human impacts from a clinical perspective without delving into molecular and cellular specificities, while other studies focus on effects triggered by specific chemicals, limiting the extrapolation of these findings to the broader impact of welding fume exposure (Aschner and Aschner 1991; Yokel and McNamara 2001; Antonini et al. 2003). Typically, exposure to these metals and their oxides triggers an immune response which can lead to disruptions in the endocrine system (Burek and Talor 2009; Sriram et al. 2014).

Despite the central nervous system’s (CNS) robust protection against external and blood-borne factors, we propose that alterations in hormonal pathways play a role in the neuroinflammatory defense mechanisms against metal oxide exposure, such as prolonged inhalation of welding fume. Changes in endocrine signalization within neurons, particularly in brain regions more sensitive to hormonal signals, may be among the first steps leading to altered cell metabolism, mitochondrial dysfunction/adaptation, and ensuing neuroinflammation (Butterfield and Halliwell 2019).

Prior research by our team and others indicates that hormonal signaling pathways are particularly vulnerable within the brain’s complex functionality (Jocsak et al. 2016, 2019; Zsarnovszky et al. 2018); however, two of the hormonal receptor groups, estrogen receptors (ERs; ERα and ERβ) and thyroid hormone receptors (TRs, TRα and TRβ), have distinct roles in the CNS. Regarding the neuroinflammatory processes, there is a quite complex counter-mechanism between these receptors. ERα and TRα is primarily involved in neuroprotection, and exerts pro-inflammatory effects (by enhancing the production of cytokines like interleukin-6 and tumor necrosis factor-alpha), which is balanced by the anti-inflammatory effects of ERβ and TRβ, particularly evident in neurodegenerative conditions like Alzheimer’s and Parkinson’s disease (Kato et al. 1995; Williams 2008; Chakrabarti et al. 2014). Furthermore, it is known that ERs and TRs interact and modulate each other’s functions, especially in the contexts of neuroinflammation and neuroprotection (Scalise et al. 2012). Considering neuroinflammation and neuroprotection, peroxisome proliferator-activated receptor γ (PPARγ) is a critical regulator of neuroprotective and anti-inflammatory processes, as well (Cai et al. 2018). The interplay between ERs/TRs and PPARγ is noteworthy, as they synergistically activate each other to exert potent anti-inflammatory effects (Hunter et al. 1996; Bonofiglio et al. 2005; Lu and Cheng 2010; Kouidhi and Clerget-Froidevaux 2018).

In summary, the interactions and regulatory patterns among ER, TR, and PPAR within the CNS are intricate and multifaceted. Their collective roles in modulating neuroinflammatory processes and neuroprotective responses are crucial, particularly in the context of environmental stressors like exposure to welding fumes. Understanding these interconnected pathways provides valuable insights into the molecular mechanisms underlying neurodegenerative diseases and the potential therapeutic targets for mitigating such conditions.

According to our working hypothesis, we suggest that specific hormone systems attempt to counteract the detrimental effects of metal oxides, such as neuroinflammation. To substantiate this hypothesis, our research focused on analyzing the transcriptional expression of key hormone receptors, including ERs, TRs, and PPARs. Our goal was to correlate the time elapsed since welding fume exposure with changes in receptor gene expression.

Our approach aims to determine whether the observed endocrine anomalies result directly from inhaled metal oxides or arise as secondary reactions to metal-induced inflammation in the brain. By determining the involvement of these pathways, we aim to provide further evidence supporting the adverse effects of welding fumes and clarify the causal link between metal oxide exposure and endocrine disruption in the CNS.

## Methods and materials

### Generation of welding fumes

To perform the fume inhalation treatments, actual welding work has been done. The welding was performed as a manual overlay weld with multiple layers on the indicated base metal plate according to the general technical requirements, and it was done by a qualified welder using a Rehm TIGER 180 AC/DC High welding machine (Rehm GmbH., Uhingen, Germany). The technical parameters for the treatment group are the following: base steel plate was EN 1.0038 according to the international standard of EN 10027–2:2015 (composition: C: 0.17, Mn: 1.40, P: 0.035, S: 0.035, Cu: 0.55, N: 0.012 m/m%, as declared by the manufacturer); Manual Metal Arc welding method was used (MMA, also known as 111 technology or “stick welding”); welding electrode was OK46.00 (ESAB, North Bethesda, Maryland, USA); electrode diameter was 2 mm; electrode polarity was DC-; welding current was 80 A. The following is the chemical composition of the welding electrode (as declared by the manufacturer)—C: 0.08, Mn: 0.4, Si: 0.3 m/m%. The coating of the electrode was rutile type.

The concentration of the fume particles was measured using an Aeroqual Model 500 instrument (Aeroqual, Auckland, New Zealand), equipped with particulate matter sensors measuring particles 10 µm or larger (PM10) and 2.5 µm and larger (PM2.5). Measurement unit is ppm. The average concentrations in the treatment chamber during the treatment for PM10 and PM2.5 were 1.32 ppm and 0.84 ppm, respectively.

### Animal experiment

Adult, pathogen-free BALB-C male, and female mice (*Mus musculus*) were used (*n* = 4 at a time) due to the absence of significant sex differences in the examined parameters. The animals were kept under a controlled 12/12-h light/dark cycle. They were fed standard chow supplied by FarmerMix Kft., Zsambek, Hungary, and had free access to tap water.

During the in vivo experiments, animals were exposed to welding fumes in treatment chambers of an EMKA Whole Body Plethysmograph system (EMKA Scientific, Paris, France). Throughout the fume exposure, the animals had free access to food and water. The airflow rate in the chambers was maintained at 1 l/min. Each treatment session lasted for 4 h daily, preceded by an acclimatization period of about 10 min. Four animals were exposed in each treatment session. The control animals were kept in the same conditions for the same time duration without exposure to the welding fumes. The animals were exterminated by cervical dislocation after the specific incubation period. Brain samples of treated and control animals were collected after 24 (early sampling) and 96 h (late sampling).

The experimental procedures were conducted under permit No. PE/EA/1335–8/2019 granted by the Animal Protection Authority of the Hungarian Government Office, adhering to all applicable guidelines and regulations, including the ARRIVE guidelines (https://arriveguidelines.org/). The Hungarian Government Office is vested with the authority to issue ethical approvals based on the Hungarian Government Decree No. 40/2013, in line with the European Union Directive 2010/63/EU. The research was carried out at the University of Veterinary Medicine in Budapest, Hungary.

### Sampling

Extermination of the animals was done with cervical dislocation, and then the cranium was opened to remove the complete brain. The brain areas of our interests were separated and kept on ice before further processing. To precisely localize the impact of welding fumes within the CNS, we focused on key brain regions based on literature and their functions, considering the entry route and primary target areas of metal oxides. The regions examined included the olfactory bulb (Oberdörster et al. 2005), hypothalamus (Dallman et al. 2005), cerebellum (Palacios et al. 2014, 2017), thalamus (Di Monte et al. 2002), hippocampus (Calderón-Garcidueñas et al. 2008), and cortex (Zatta et al. 2003).

### Quantitative-RT-PCR

Total RNAs were isolated from brain tissue samples with TRI reagent as per the manufacturer’s guidelines (Invitrogen, Carlsbad, CA, USA), and further purification was accomplished using the Direct-zol RNA Miniprep kit (Zymo Research, Irvine, CA, USA). The quantification and assessment of RNA purity were performed spectrophotometrically using a NanoDrop™ ND-1000 device (Wilmington, NC, USA), focusing on absorbance ratios at 260–280 nm (Wilfinger et al. 1997). Subsequently, 3 μg/μL of the total RNA was reverse transcribed in an RT-PCR process (Amplitron II., Barnstead/Thermolyne, Dubuque, IA, USA) utilizing M-MLV reverse transcriptase, oligo (dt) primers, and a dNTPmix.

For qRT-PCR analysis, 2 μL of the synthesized cDNA was employed in triplicate, using the Master SYBRGreen method (F. Hoffmann-La Roche, Basel, Switzerland) on a LightCycler 2.0 (F. Hoffmann-La Roche, Basel, Switzerland). Glyceraldehyde 3-phosphate dehydrogenase (GAPDH) was utilized as a reference gene for normalization purposes (Bustin 2002). Primer design was based on NCBI’s Primer-BLAST tool or adapted from existing literature, with a final concentration of 2 µM for each primer pair. The primer sequences for GAPDH, ERα, ERβ, TRα, TRβ, and PPARγ are detailed in Jocsak et al. (2019) (Table 1). PCR cycling parameters and controls were aligned with the manufacturer’s recommendations and optimized according to Jocsak et al. (2016). Analysis of real-time PCR threshold cycle (Ct) data was performed using REST-XL software version 2.0 (GenEx-BioMcc, TUM, München, Germany) (Pfaffl et al. 2002). Ct values were normalized against GAPDH, and relative mRNA expression ratios (fold changes) were computed employing the 2^−ΔΔCt^ method (Livak and Schmittgen 2001).

**Table 1:** Primer sequences used for qRT-PCR analysis

Target gene (mouse)	Primer sequence 5′–3′	
ERα	Forw	GGA ACT GTC TGC CCA TCG TT
	Rev	GAA CCC AGG GCT GCC TTA C
ERβ	Forw	AAC CTT CCT CTT GGG CAT CG
	Rev	TTT CAT CCG GTT CTC CCA CC
TRα	Forw	ACC GCA AAC ACA ACAT TCC G
	Rev	GGG CCA GCC TCA GCT AAT AA
TRβ	Forw	CGA GGC CAG CTG AAA AAT GG
	Rev	CTC AGC ACA CTC ACC TGA AGA
PPARγ	Forw	TTG GTG GGA TTG TGT CTC GG
	Rev	GGC CAA GAT CTC ACA GTG CT
GAPDH	Forw	TGA AAT GTG CAC GCA CCA AG
	Rev	GGG AAG CAG CAT TCA GGT CT

### Data analysis

Following the determination of normality distribution by the Shapiro–Wilk test, two-way ANOVA with Bonferroni post-tests was used according to the guidance of the Department of Biomathematics, University of Veterinary Medicine, Budapest, Hungary. All statistical analyses were conducted using GraphPad Prism version 9 (GraphPad Software, San Diego, CA, USA). Results are presented as mean ± standard deviation (SD). All statistical tests were two-tailed, and *p* values less than 0.05 were considered statistically significant.

## Results

Our findings are organized by receptor families (ER, TR, and PPAR), showcasing the expression levels of these receptors across various brain regions (OB, HT, CE, T, HC, and C). The data is presented in a comparative format, juxtaposing the values for early and late sampling periods side by side. The data from each brain region and sampling period is compared to their respective control, and fold changes are shown on the graphs. The control groups (always 1) are not included in the figure.

In general, the examined receptor expressions show a similar pattern. The early sampling (24 h) mostly caused minor adjustments only presenting downregulation in most brain regions. On the other hand, late sampling (96 h) revealed more prominent changes in all brain regions. Interestingly, these changes show downregulation in most of the brain parts except for the olfactory bulb and the hypothalamus. In the latter regions, we detected an evident upregulation in examined receptors (except ERα).

Due to a deeper understanding, we present our most intriguing findings at each receptor separately.

### Changes in estrogen receptor mRNA levels after welding fume exposure

**Fig. 1:**
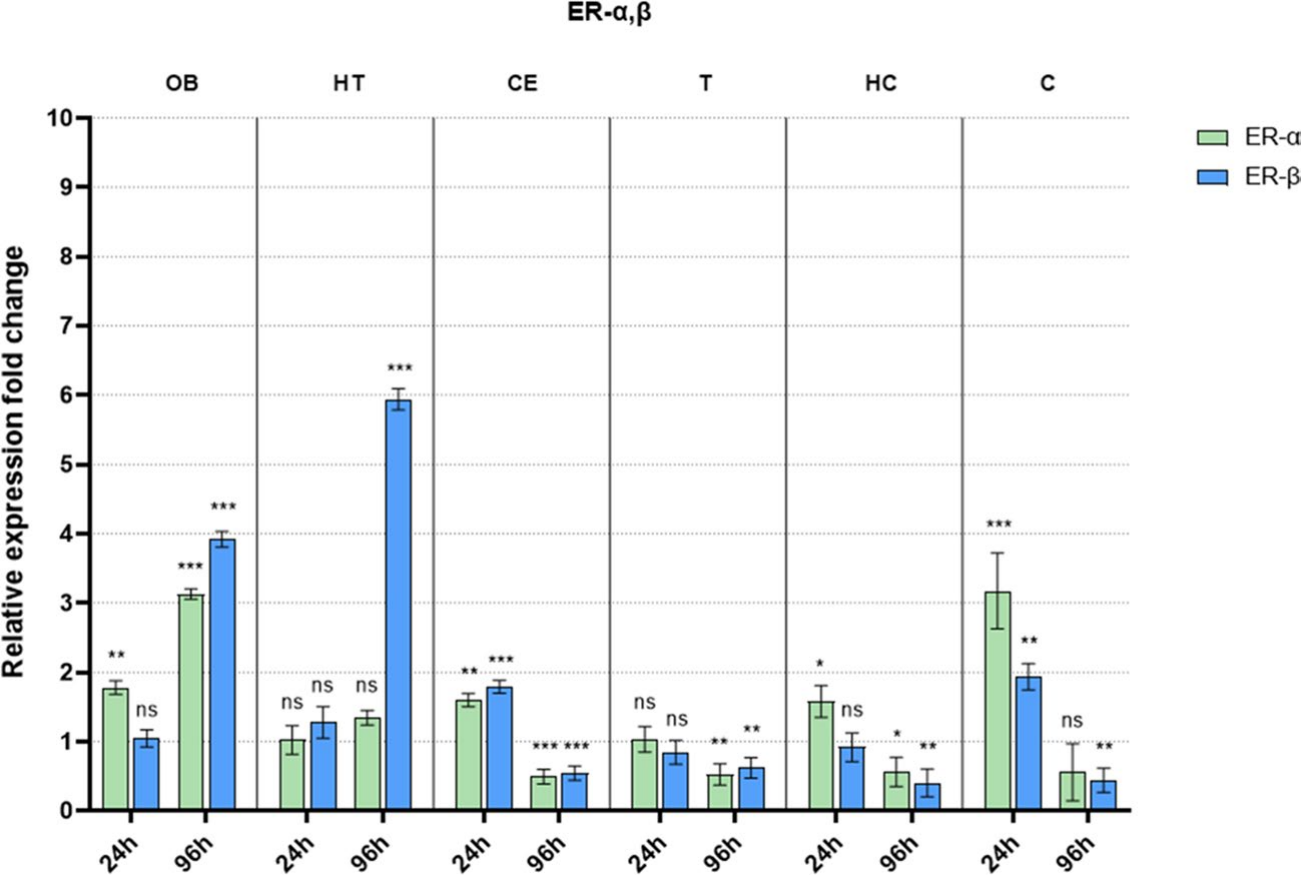
Fold difference in estrogen receptor mRNA levels after welding fume exposure compared to their respective controls (not shown in the figure) in all examined brain regions following early (24 h) and late (96 h) sampling (OB: olfactory bulb; HT: hypothalamus; CE: cerebellum; T: thalamus; HC: hippocampus; C: cortex; ns: not significant compared to its respective control; significance: *p < 0.05, **p < 0.01, and ***p < 0.001)

Changes in ERs are shown in Fig. 1. In the OB, early sampling already resulted in an elevation of ERα receptor expression, whereas ERβ levels remained close to baseline. Late sampling revealed a further increase in ERα. This increase, however, was followed by a significant rise in ERβ, outpacing the changes observed in ERα. In HT, ERβ showed a very dominant change in late sampling only. Another finding that stands out of the general expression pattern observed at all receptors is found in the cortex (*C*). Here the early exposure caused a considerable increase in both receptors, while late sampling presents a downregulation. This pattern was mirrored in the CE and HC regions at a smaller scale.

### Changes in thyroid receptor mRNA levels after welding fume exposure

**Fig. 2:**
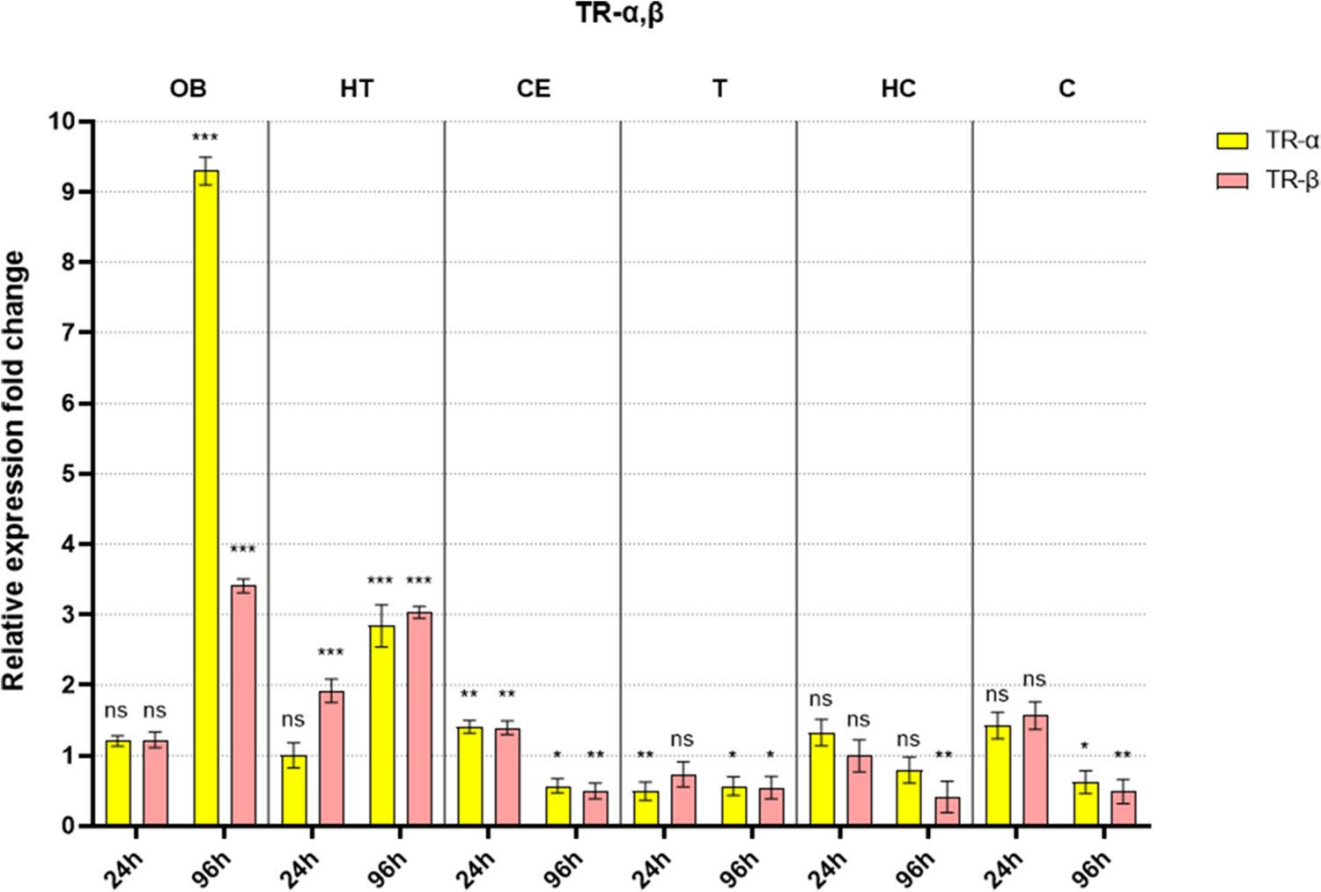
Fold difference in thyroid receptor mRNA levels after welding fume exposure compared to their respective controls (not shown in the figure) in all examined brain regions following early (24 h) and late (96 h) sampling (OB: olfactory bulb; HT: hypothalamus; CE: cerebellum; T: thalamus; HC: hippocampus; C: cortex; ns: not significant compared to its respective control; significance: *p < 0.05, **p < 0.01, and ***p < 0.001)

Changes in TRs are shown in Fig. 2. Thyroid receptors displayed a brain region-specific transcriptional pattern similar to estrogen receptors. One of the major differences is that we can observe a more intense change in OB and a less intense in HT and in the case of TRß upregulation already starts at early sampling. Still, the directions of these visible changes are the same as those detected in ER groups. Also, the early sampling of the other regions does not show any upregulation, but still, they are followed by a downregulation at late sampling.

### Changes in PPARγ mRNA levels after welding fume exposure

**Fig. 3:**
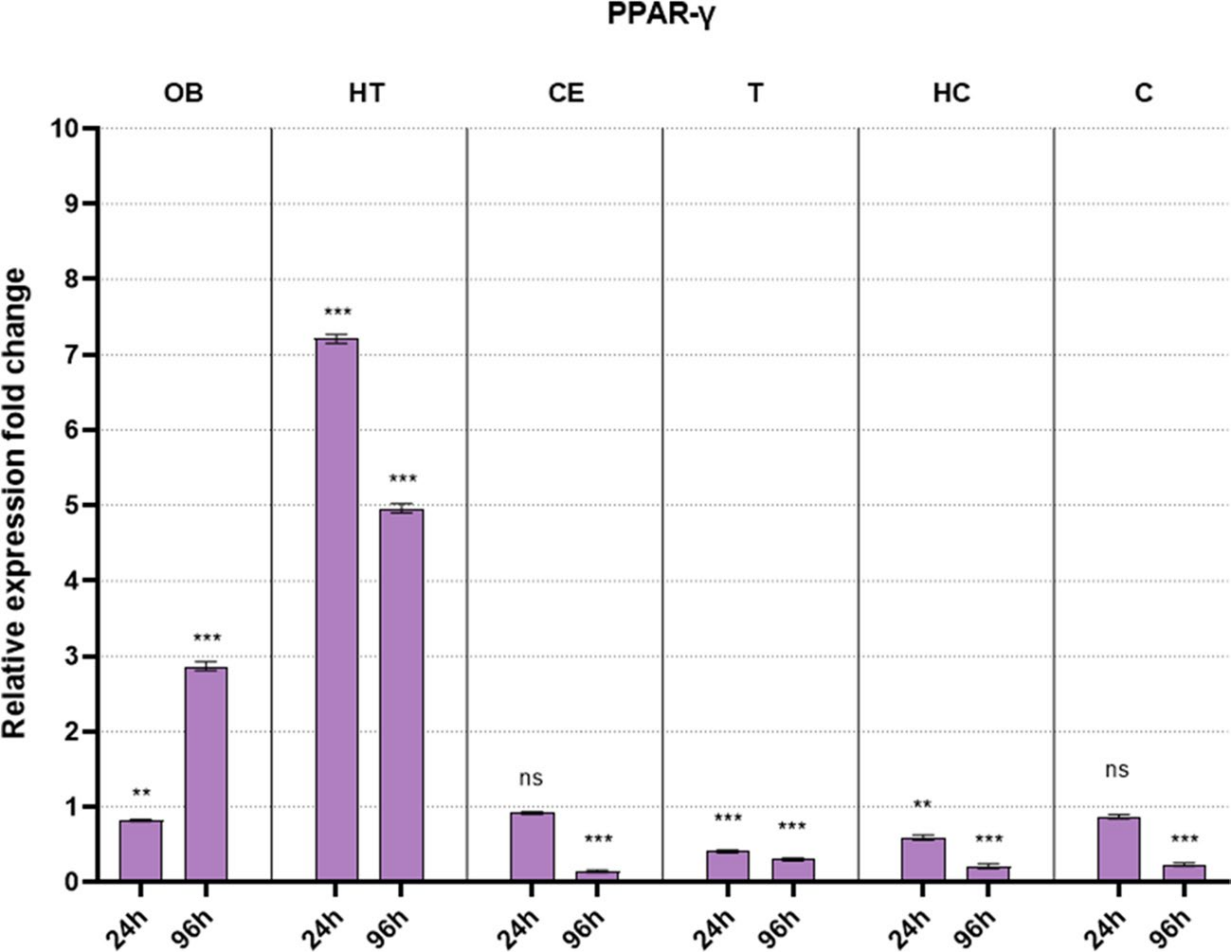
Fold difference in PPARγ mRNA levels after welding fume exposure compared to their respective controls (not shown in the figure) in all examined brain regions following early (24 h) and late (96 h) sampling (OB: olfactory bulb; HT: hypothalamus; CE: cerebellum; T: thalamus; HC: hippocampus; C: cortex; ns: not significant compared to its respective control; significance: *p < 0.05, **p < 0.01, and ***p < 0.001)

Changes in PPARγ are shown in Fig. 3. PPARγ receptor expression mirrored the trends seen in thyroid receptors, with late upregulation in OB and HT, and late downregulation in all other areas, also downregulation starts early in T and HC. There is only one substantial difference compared to the other receptors examined: in HT upregulation starts already after 24 h, and it is even stronger than the one found in late sampling.

## Discussion

Welding fumes, comprising a complex mixture of particles and gases, including metals and oxides, pose significant risks to the nervous system. The harmful characteristics vary with the welding technique. The impact of metals and metal oxides on endocrine events in the CNS is intricate. It is well-established that heavy metals such as lead, mercury, and cadmium can disrupt endocrine signaling (Sanders et al. 2009). Also, Mn affects the CNS and potentially disrupts hormonal pathways (Santamaria 2008), while iron overload can impair the pituitary gland (Moos et al. 2007) and also capable to modify the pro- versus anti-inflammatory functions of the microglia cells (Nnah and Wessling-Resnick 2018).

The disruption of the endocrine system is easily backed by our findings: the most prominent changes are found in the hypothalamus (HT) and olfactory bulb (OB). This is not surprising after all: OB is considered the first target of welding fumes (Ngwa et al. 2014; Sriram et al. 2014) due to its links to the nasal cavity. HT, one of the main regulators of many physiological processes, has a weakened BBB (fenestrated capillaries), which allows a faster response in regulatory processes, but at the same time, it makes it more vulnerable to harmful stimuli (Haddad-Tóvolli et al. 2017). The other brain regions are seemingly more protected from the absorbed components of welding fumes as we found only minor (or no) changes after 24 h, as a result of protection that is most probably due to the BBB. On the other hand, this shield of the neurons is not able to repel all toxic ingredients of welding fume as all brain areas are showing trends of receptor downregulation in all late sampling groups.

By analyzing the expression patterns of late sampling, we can find a strong upregulation in OB and HT while a downregulation in the other regions. These opposite results can also be explained by the anatomical differences in the BBB and indicate a dose-dependent mechanism of action already found in vitro (Vettori et al. 1999; Konoha et al. 2006). This difference raises the possibility that some brain areas might be less vulnerable to the rapid action of single exposure, however, the receptor downregulation cannot be fully explained. A long-term experiment and further parameters examined could clarify this enigma, as well as the mechanisms behind cortical, hippocampal, and thalamic dysfunctions as results of welding fume exposure (e.g., impairment of fine motor control and cognitive dysfunctions) (Kenangil et al. 2006; Sriram et al. 2010; Verhoeven et al. 2011; Stamelou et al. 2012).

In summary, these results showing dose- and region-dependent changes further deepen our understanding of the synergistic distributing effects of a mixture of harmful materials. But when focusing on the individual receptors, a different pattern is also emerging. The examined receptors are not exclusive to the endocrine system, but they can be found in most cell types exerting a complex effect ranging from cellular development to metabolic processes and inflammation.

The neuroinflammatory impact of welding fumes (a composite of metals and metal oxides) is far from well-documented. One notable study reported increased expression of proinflammatory chemokines and cytokines in a rodent model after short-term mild steel welding fume exposure (Antonini et al. 2009). Our primary goal in this study was to identify possible hormonal disruption effects of the welding fumes. However, our results revealed a complex interplay between the receptors which is potentially able to counteract the inflammatory effects induced by welding fume exposure as these receptors collectively orchestrate a series of neuroprotective and anti-inflammatory responses within various brain regions (Williams 2008; Vegeto et al. 2008; Heneka et al. 2010). In the next paragraphs, we try to shed some light on these mechanisms by focusing on the results of the individual receptors and their interactions.

Our results revealed an increase in ERs, particularly ERβ, in HT and OB. The simultaneous activation of ERß and ERα indicates strong neuroprotective mechanisms (Simpkins et al. 2012). The acute increase in ERα in the OB and subsequent elevation of both ERα and ERβ in HT align with the estrogenic role in mitigating inflammatory response, a common consequence of metal exposure in welding fume (Antonini et al. 2009). In our research, probably, the excess upregulation of ERß could be attributed to the pro-inflammatory action of ERα balancing the neuroprotective mechanisms promoting cellular survival and repair mechanisms.

The TRs show a similar pattern of increased expression in OB and HT that implies active involvement in modulating the brain’s response to the welding fumes. TRα is responsible for the classical functions of thyroid hormone, such as metabolism and energy homeostasis; however, from a neural point of view, its importance lies in compensating for fluctuations in the energy supply of the brain tissue (Williams 2008; McAninch and Bianco 2014). Its upregulation represents a compensatory role in maintaining energy homeostasis under stress conditions, such as those induced by neuroinflammation. On the other hand, TRß is also involved in neuroinflammation as it can suppress inflammatory responses (Williams 2008). However, the exact mechanisms underlying these effects are still being investigated; the co-activation of both TRs in our research suggests some kind of counter-balancing mechanism of the two subtypes.

PPARγ activation in the OB and HT also suggests the initiation of neuroprotective mechanisms, possibly countering neuroinflammation induced by metal accumulation (Racette et al. 2012). The most intriguing finding is the early increase of PPARγ in HT. Such an early response is absent with regard to the other receptor types. This result does not fit into the general pattern discussed above but can be easily explained if we consider that the hypothalamic cells are strongly involved in the energy metabolism of the whole body (Timper and Brüning 2017). PPARs are key factors in intracellular metabolisms; therefore, they must be even more prone to change in the hypothalamic environment.

Considering the similarities in PPARγ, ER, and TR results, it is reasonable to assume that the pathways are interconnected to potentially amplify each other’s neuroprotective effects, pointing towards a synergistic mechanism. This collaborative response likely involves the modulation of inflammatory processes, reduction of oxidative stress, and promotion of neuronal survival and repair. The increased expression of these receptors suggests an attempt by the CNS to initiate a protective response against the neurotoxic effects of welding fumes, particularly those associated with Mn and Fe exposure.

From all welding fume ingredients, mainly Mn has a well-known role in neuroinflammation, but some evidence links the other components to the inflammatory processes, as well. Manganese accumulation in the CNS leads to oxidative stress and pro-inflammatory cytokine release, contributing to neurodegenerative diseases in metal workers (Erikson and Aschner 2003; Racette et al. 2012), excess iron in the brain, notably in the substantia nigra, triggers a neuroinflammatory cascade (Ward et al. 2014).

The manganese penetration pathway into the CNS is still under investigation. However, the most probable hypothesis is the transport via the BBB; there are data about the direct Mn absorption via the OB from the nasal cavity (Tjälve et al. 1996; Salehi et al. 2003). Our results also support the theory of manganese penetration to the CNS via the lung-blood-BBB pathway. As it was shown, the receptor gene expression rates in the OB react always later than the same expression rates in the HT (96 h vs. 24 h). According to this fact, it seems plausible that the Mn enters the CNS via the BBB into the HT; however, the BBB is weakened here by fenestrated capillary structure. The gene expression rate changes in the C region seem to be in contradiction with this theory since we were able to demonstrate early gene expression changes after the treatment. In our view, this phenomenon must be the result of some underlying cascade functions, which are yet to be discovered.

To address the limitations of this study, it is important to note that we conducted a screening experiment focusing only on specific brain regions and limited our investigation to a selected few hormone receptors. This choice was influenced by the scarcity of literature on metal oxide and welding fume exposure specifically targeting endocrine functions, as well as the lack of separate studies examining various brain regions. Additionally, it is crucial to acknowledge that fumes from different sources or compositions may induce varying, organ- or even and tissue-specific effects. Moreover, the duration of exposure is likely to influence outcomes, necessitating future research on chronic impacts to provide a more comprehensive understanding of real-world scenarios. We believe that our pioneering study offers initial insights into optimizing sampling times across different organs. Lastly, an inherent limitation is the use of animal models, as rodents may react differently due to macroscopic differences in respiratory tract structure compared to humans.

## Conclusion

In summary, our study shows a complex neurophysiological mechanism of welding fumes, highlighting brain region-dependent interactions with hormone receptors. The hypothalamus and olfactory bulb show significant receptor alterations, underscoring their vulnerability and regulatory roles, particularly in the context of a weakened blood–brain barrier. These findings deepen our understanding of how welding fume exposure, including metals like manganese and iron, disrupts endocrine signaling and potentially exacerbates neuroinflammation.

The observed changes in receptor expressions suggest a neuroprotective response, potentially mitigating inflammatory effects despite initial vulnerability. Obviously, further research will be essential to fully uncover these mechanisms and assess long-term implications. Understanding the complex interactions and regulatory mechanisms of these receptors in response to environmental stressors like welding fumes could pave the way for developing targeted therapeutic strategies to combat neuroinflammation and related neurodegenerative conditions related and unrelated to welding.

## Data Availability

Not applicable.

## Declarations

### Disclaimer

The founding sponsors had no role in the design of the study; in the collection, analysis, or interpretation of data; or in the writing of the manuscript.

### ARRIVE guidelines

The authors have read the ARRIVE guidelines, and the manuscript was prepared and revised according to the ARRIVE guidelines.
